# Intelligence

**Published:** 1807-10

**Authors:** 


### INTELLIGENCE.

Government having purchased the Museum of the late JOHN HUNTER, Esq. whose choice and rare Collection of natural and artificial Curiosities are so very peculiarly qualified to serve the science of Surgery, have thought it expedient to convert the whole of it to the public benefit, by presenting the same to the Royal Corporation of Surgeons: for which purpose a commodious and extensive building is erecting in Lincoln's-inn-square, where this valuable and splended assemblage will be deposited, and where, within one extensive area, will be at once concentred, the Corporation Hall, the Anatomical Theatre, the Repository of Curiosities, and the several apartments and Chambers of the Officers of the Corporation. The grand front will be towards Portugal-street. The whole structure will be completed early in the ensuing spring, and present a splendid edifice.

SIR JOSEPH BANKS has recently stated the advantages to be obtained by inuring tender plants, natives of warmer climates, to bear the severity of that of England: In the case of annuals, he mentions this is effected with little trouble, as all that is required is to enable them to ripen their seed in a comparative cold summer, after which the hardest frost will have no power to injure it: but a perennial has to encounter frosts with its buds and annual shoots, that have sometimes been so severe with us as to render asunder the trunks of our indigenous forest trees. In 1791, some seeds of Zizania aquatica were procured from Canada, and sown in a pond at Spring Grove, near Hounslow. They grew, and and produced strong plants, which ripened their seeds. These vegetated in the succeeding spring, and so on evey year, the plants springing up from the seeds of the preceding year, and becoming visibly stronger and larger, and rising from deeper parts of the pond, till the year 1804, when several of the plants were six feet high, and the whole pond was in every part covered with them. From this and other similar experiments, Sir Joseph proposes to sow the seeds of such shrubs as occasionally ripen them in the English climate, after the example of the Zizania, which, in fourteen years, became completely naturalized to our climate.

M. Lechenault, one of the Naturalists belonging to the expedition under Captain Baudin, who was detained at Batavia by illness, has just arrived at Nantz with a superb collection of Natural History, and one of the most valuable collections of East India arms and instruments, procured from Otaheite, Java, and the adjacent islands. He has neglected no means of enriching Natural History, by furnishing several kinds hitherto unknown.

The Josephine Academy of Surgery, at one of its late meetings, was principally engaged in a careful examination of the conductor of light, invented by Dr. Bozzini, of Frankfurt on the Mayn, and which is intended to illumine the internal parts and cavities of the body. The experiments, which were made on corpses, were attended with results highly houourable and satisfactory to the inventor, and fully demonstrated the utility of this ingenious contrivance. It is more than doubtful, however, that the application of this conductor of light to living persons will be attended with very great dtfficulties.

---

### St. George's Hospital.

Mr. Home's Lectures on the Principal Operations in Surgery, given gratuitously to the pupils of this Hospital, will commence in October next, as usual.

Mr. Gunning, Surgeon to St. George's Hospital, will commence his Lectures on the Principles and Operations of Surgery, on Monday the 5th of October next, at eight o'clock in the evening.——Particulars may be known at Mr. Gunning's house, 45, Conduit Street, Hanover Square, or at St. George's Hospital.

---

Dr. Clough, Physician and Man-midwife to the St. Mary-le-bone General Dispensary, &c. will, on Monday, the 5th of October, at ten o'clock in the morning, commence his Course of Lectures on the Theory and Practice of Midwifery, and on the Diseases of Women and Children. Further particulars may be known on application to Dr. Clough, No. 68, Berner's Street; Mr. Wilson, Medical Agency Office, No. 56, St. Paul's Church Yard; and at the Dispensary, No. 77, Welbeck Street.——N. B. An Evening Course, for the accomodation of such Students as cannot attend the Morning Lectures, will be given on Tuesdays, Thursdays, and Saturdays, during the Winter, at seven o'clock.

Dr. Hooper will commence a Course of Lectures on the Theory and Practice of Physic, the Materia Medica, and Pharmaceutical ceutical Chemistry, on Monday, the 12th of October, at his Lecture Room, in Cork Street, Burlington Gardens, at eight o'clock in the morning.

Mr. Moor, Surgeon Dentist to her Royal Highness the Duchess of York, will commence a Course of Lectures on the Structure and Diseases of the Teeth, on the 5th of November, in which will be explained the Complete Practice of the Dentist. Further particulars may be known at his house, No. 6, Palsgrave Place, Temple.

Dr. Reid's Course of Lectures on the Theory and Practice of Medicine will recommence, on Monday, the 5th of October, at eight o'clock in the evening, at his house in Grenville Street, Brunswick Square; at which place the ensuing Lectures will be delivered at ten o'clock on the mornings of Mondays, Wednesdays, and Fridays.

Mr. Taunton will commence his Autumnal Course of Lectures on Anatomy, Physiology. Pathology, and Surgery, on Saturday, October 3, 1807, at eight o'clock in the evening, at his house in Greville Street, Hatton Garden. The Lectures will be continued every Tuesday, Thursday, and Saturday, at the same hour.

M. Alibert, Physician of the Hospital of St. Louis, has commenced a work on Disorders of the Skin. The second number relates to that dreadful disorder the *Plica*, of which he enumerates five sorts. He describes their general and particular symptoms, their analogy with the disorders, and the causes favourable to their production, Five instances have been found in Paris ; he has gathered his information from these, and from an extensive correspondence with Polonese physicians.

Mr. Accum, Lecturer on Practical Chemistry and Mineralogy, has begun to print A System of Mineralogy and Mineralogical Chemistry, and its Application to the Arts. The work is formed chiefly on the plans of Stacey and Brongniart, and will be published in 2 vols. 8vo. with eight copper-plates.

Dr. Beddoes, has in contemplation, a Medical Work of great extent, comprising a Collection of Facts, made by the Original Observers of Fever in all Nations.

The Election of Medical Officers to the London Female Penitentiary, took place, by ballot, on Friday, Sept. 11, when there were ten candidates. The majority of votes were in favour of Dr. Pinckard as Physician; Mr. Blair as Surgeon; and Mr. S. Griffith as Apothecary.

